# Population dynamics of the human gut microbiome: change is the only constant

**DOI:** 10.1186/s13059-019-1775-3

**Published:** 2019-07-31

**Authors:** Sambhawa Priya, Ran Blekhman

**Affiliations:** Department of Genetics, Cell Biology, and Development, University of Minnesota, Minneapolis, MN 55455 USA

## Abstract

Characterization of the temporal dynamics of the human gut microbiome is crucial for understanding its role in modulating host health. Two recent studies explored the genetic diversity of gut microbes and unraveled extensive longitudinal dynamics within the host that is driven by natural selection.

## Introduction

Our gastrointestinal tract harbors a complex ecosystem of trillions of microbial cells, called the gut microbiome, which plays a critical role in maintaining our health. Elucidation of the ecological and evolutionary dynamics of the gut microbiome is essential for determining its effect on host health and disease. Longitudinal studies have reported long-term stability of the high-level taxonomic composition of gut microbiota communities [1, 2]. However, population-level analyses of gut bacteria have revealed a high rate of strain-level turnover with timescales of months to years [3, 4]. Given this turnover, the genetically diverse resident clonal populations might be subject to evolutionary forces that shape adaptation within the microbial community and impact their long-term persistence in the gut, thereby influencing host health. We know very little about how these evolutionary forces operate at the population level within the healthy human gut microbiome.

A few cultivation-based longitudinal studies have shown temporal diversity in the resident strains of the gut microbiome [4]. These population-level dynamics could alter gut microbiome function, and could determine whether a strain will be transient or whether it will persist and colonize the gut. However, there are very few estimates of residency (i.e., the length of time that a microbe occupies a niche in the gut) that can be used to identify long- and short-lived members of the human gut microbiome. To address this, Martinson et al. [5] examined gut microbiome residency in healthy individuals and found substantial variability within the resident clonal populations of the healthy gut microbiome.

Given this genetic diversity at the population level, commensal bacteria might have the opportunity to evolve and adapt within the human gut. Previous studies have shown the adaptive evolution of microbial species in the context of infection [6] or laboratory experiments [7], but we do not know whether and how genomic adaptations occur within the complex ecosystem of the healthy human gut. To address this, Zhao et al. [8] characterized the population dynamics of a commensal to identify signatures of adaptive evolution within a healthy gut ecosystem.

### Population-level dynamics defy community-level stability of *Enterobacteriaceae* in the healthy human gut microbiome

Martinson et al. [5] quantified and compared the community- and population-level diversity within the human gut microbiome to identify static and dynamic elements. To do so, they collected fecal samples once every 2 weeks over a span of 17 months from eight healthy human adults and characterized the community-level dynamics by quantifying the abundance and residency of taxa (defined as operational taxonomic units and amplified sequence variants) using 16S rRNA sequencing. In addition, using selective culturing and PCR-based genotyping on the same fecal samples, they evaluated the population-level dynamics of the resident clones of *Enterobacteriaceae*, a common and taxonomically diverse family of gut bacteria. Their analysis revealed population-level turnover of *Enterobacteriaceae* resident clones over a short time-period (months to years), which contrasted with the observed stability at higher taxonomic levels over time.

Using beta-diversity analysis on taxonomic composition, they found that the longitudinal fecal samples clustered by patients, irrespective of the large differences in sampling time, thus confirming the community-level stability of the microbiome over time. In addition, Martinson et al. [5] investigated gut microbiome residency by calculating the time difference between the first and last observation of a taxon in a fecal sample. They found that significant proportions of the taxa were mostly transient throughout the duration of the study period, and that there was little evidence of a ‘core resident microbiome’ (i.e., a set of resident taxa that were common across subjects). In addition, a culture-based approach to identify the occurrence of distinct clonal lineages of *Enterobacteriaceae* over time revealed variability in residence times. Out of 32,470 single colonies, a total of 120 unique *Enterobacteriaceae* clones were identified across all the subjects, and only 31% of these were found to be resident. Among these, *Escherichia coli* was found to be the most dominant resident species, and specific phylogroups of *E. coli* (A, B2.3, and F) were more likely to be human residents over longer periods of time than were other *Enterobacteriaceae* lineages. Thus, this study demonstrates that high-level taxonomic analyses fail to capture the population-level dynamics and short-term turnover of *Enterobacteriaceae* clones in the human gut microbiome.

### Rapid and host-specific adaptive evolution of *Bacteroides fragilis* in the healthy human gut

The longitudinal diversity observed in the resident strains might be shaped by evolutionary forces acting at the population level within the microbiome. To investigate this, Zhao et al. [8] combined culture-based population genomics and metagenomics to characterize the within-host evolutionary dynamics of a prevalent gut commensal, *Bacteroides fragilis*, in healthy humans. To do so, they sequenced the whole genomes of 602 *B. fragilis* isolates cultured from longitudinal and cross-sectional fecal samples collected from 12 healthy individuals. Using the draft genomes of the isolates, they identified single nucleotide polymorphisms and built phylogenies which revealed that each subject was colonized by a single lineage of *B. fragilis*. Moreover, this lineage diversified within the host to form coexisting sublineages. For most lineages, the time to most recent common ancestor was found to be between 1.1 to 10 years before initial sampling, suggesting that the sublineage diversification occurred within each subject’s lifetime. The resulting estimates of divergence time were substantially smaller than each subject’s age, implying either late colonization or early colonization with a neutral or adaptive sweep of a single sublineage. By identifying genomic regions that had high average relative coverage in the metagenomes compared to the isolate genomes, the authors also found mobile elements, such as prophages, integrative conjugative elements, and plasmids, that transfer within an individual’s microbiome, contributing to the within-host evolution of *B. fragilis*.

Next, the authors examined whether within-lineage mutations in *B. fragilis* were under positive selection. They identified 16 genes in which the recurrent mutations were concentrated and found that these genes were enriched for amino-acid-changing mutations compared to amino-acid-preserving mutations, suggesting that these genes had evolved under positive selection. Moreover, these genes are involved in cell-envelope biosynthesis and outer-membrane polysaccharide utilization, suggesting that they might be adapting to protect the microbes against phage predation or to modify the interaction with the host immune system in order to maximize the survival of *B. fragilis* in vivo. The majority of the identified genes had mutations that appeared in a single host, suggesting that the selective forces shaping bacterial genomes within the microbiome can be host-specific. Interestingly, the same genes that were found to evolve under positive selection within hosts were also found to evolve under purifying selection across lineages, suggesting that diverse selective forces act within and across hosts. In considering an amino acid change that had high incidence across the study subjects, the authors used publicly available data to investigate the frequency of this mutation across human populations. They found that the amino acid is frequently mutated in the gut microbiomes of individuals from the US and UK, but not in those of individuals from China, alluding to a selective pressure that is population-specific. This study showed a rapid adaptation that is specific to *B. fragilis*, whereas another recent study, which used metagenomics to characterize evolutionary dynamics across species, reported evidence of within-host adaptation, implying the presence of microbiome-wide signatures of adaptive evolution [9].

### Implications and future directions

The ecological and taxonomic properties of the human gut microbiome have been extensively characterized, but the evolutionary dynamics within the microbial population of the human gut are not well understood. Recent studies that have shed light on the population dynamics and microbial evolution within the healthy human gut have advanced our understanding of how microbiomes affect host health. Within-host adaptive evolution of the microbiome could potentiate long-term persistence and colonization in the gut, and may have an effect on the microbiome’s resilience to pathogenic invasions. These findings motivate questions for future studies: how are the selective pressures that drive microbial adaptation affected by host-specific factors, such as diet, medication use, and host genetics? How is the evolution of a particular microbial strain affected by factors related to the microbiome community in which it resides, such as inter-microbial interactions, metabolism, and spatial organization? How do these microbial genetic adaptations affect the host, and through what mechanism?

**Fig. 1:**
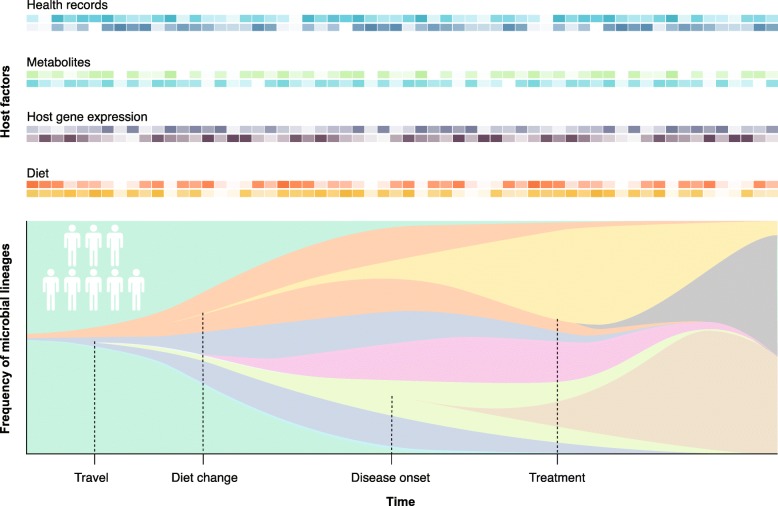
Tracking evolutionary dynamics in the human gut microbiome over time. Longitudinal studies tracking a large number of individuals, including relevant host factors, such as diet, gene expression, metabolites, and health records (*top*) will be necessary to provide an understanding of the evolutionary dynamics of the human gut microbiome throughout a person’s life (*bottom*)

Unraveling the nature and specificity of the evolutionary forces that act on members of the human gut microbiome are integral to leveraging the microbiome to improve human health. Doing so would require longitudinal studies tracking large numbers of individuals, including relevant host factors such as diet, health status, medication use, weight gain or loss, travel, socio-economic factors, and host genetics (Fig. 1). In addition, the integration of additional genomic and health data would help to uncover the mechanisms through which these dynamics impact the host. Culture-free approaches for the accurate detection of species-specific de novo mutations, such as long-read sequencing, are needed to scale up the population genomics framework to the whole-community level. Last, in vitro and in vivo model study systems, such as organoids or mouse models, can be used to reveal the causal effects that microbial mutations have on host phenotypes and health [10]. A better understanding of the selection pressures driving evolution within the microbiome will guide the choice or engineering of microbial strains for long-term colonization, thus facilitating the development of effective microbiome-based therapeutics.
